# Validation of a theoretically motivated approach to measuring childhood socioeconomic circumstances in the Health and Retirement Study

**DOI:** 10.1371/journal.pone.0185898

**Published:** 2017-10-13

**Authors:** Anusha M. Vable, Paola Gilsanz, Thu T. Nguyen, Ichiro Kawachi, M. Maria Glymour

**Affiliations:** 1 Center for Population Health Sciences, Department of Medicine, Stanford University, Palo Alto, California, United States of America; 2 Center for Primary Care and Outcomes Research, Department of Health Research and Policy, Stanford University, Palo Alto, California, United States of America; 3 Department of Social and Behavioral Sciences, Harvard T. H. Chan School of Public Health, Harvard University, Boston, Massachusetts, United States of America; 4 Department of Epidemiology and Biostatistics, University of California, San Francisco, San Francisco, California, United States of America; 5 Division of Research, Kaiser Permanente, Oakland, California, United States of America; University of Bristol, UNITED KINGDOM

## Abstract

Childhood socioeconomic status (cSES) is a powerful predictor of adult health, but its operationalization and measurement varies across studies. Using Health and Retirement Study data (HRS, which is nationally representative of community-residing United States adults aged 50+ years), we specified theoretically-motivated cSES measures, evaluated their reliability and validity, and compared their performance to other cSES indices. HRS respondent data (N = 31,169, interviewed 1992–2010) were used to construct a cSES index reflecting childhood social capital (cSC), childhood financial capital (cFC), and childhood human capital (cHC), using retrospective reports from when the respondent was <16 years (at least 34 years prior). We assessed internal consistency reliability (Cronbach’s alpha) for the scales (cSC and cFC), and construct validity, and predictive validity for all measures. Validity was assessed with hypothesized correlates of cSES (educational attainment, measured adult height, self-reported childhood health, childhood learning problems, childhood drug and alcohol problems). We then compared the performance of our validated measures with other indices used in HRS in predicting self-rated health and number of depressive symptoms, measured in 2010. Internal consistency reliability was acceptable (cSC = 0.63, cFC = 0.61). Most measures were associated with hypothesized correlates (for example, the association between educational attainment and cSC was 0.01, p < 0.0001), with the exception that measured height was not associated with cFC (p = 0.19) and childhood drug and alcohol problems (p = 0.41), and childhood learning problems (p = 0.12) were not associated with cHC. Our measures explained slightly more variability in self-rated health (adjusted R^2^ = 0.07 vs. <0.06) and number of depressive symptoms (adjusted R^2^ > 0.05 vs. < 0.04) than alternative indices. Our cSES measures use latent variable models to handle item-missingness, thereby increasing the sample size available for analysis compared to complete case approaches (N = 15,345 vs. 8,248). Adopting this type of theoretically motivated operationalization of cSES may strengthen the quality of research on the effects of cSES on health outcomes.

## 1 Introduction

Childhood socioeconomic status (cSES) is a powerful predictor of later life health outcomes [1], via pathways which appear to be distinct from the effects of adult SES [2–5]. Partially because SES is a complex social phenomenon and it may not be possible to capture it with a single measure, the operationalization of cSES has varied substantially across studies, and even between analyses within the same data set [6–8]. For example, in analyses using data from the Health and Retirement Study, indicators for cSES have been created using both parents’ education [9], residential mobility for financial reasons during childhood [10], and combinations of parental education, occupation, and retrospectively reported SES [3,4,11]. Theories of social stratification offer alternative frameworks for conceptualizing cSES, such as human capital theory, social production of disease theory, or the theory of fundamental causes [12–15], however, few researchers investigating cSES specify an explicit conceptual framework to motivate their interest in social stratification or their operationalization of cSES.

Lack of an explicit conceptual framework for cSES has implications for inference. First, when cSES is used to control for confounding of other risk factors, proxy indicators for cSES may not capture all the relevant dimensions, resulting in residual confounding by cSES [14,16]; this can occur when, for example, cSES is operationalized by father’s occupation alone, and relevant dimensions such as mother’s education are excluded, resulting in residual confounding by mother’s education. This residual measurement error can be particularly problematic when investigating the role of cSES in racial or geographic disparities, potentially leading to an underestimation of the contribution of cSES in such disparities [15,17]. Second, a well-defined treatment is one of the assumptions of causal inference [18]; without a well-defined exposure variable in observational studies, policy interventions to ameliorate the impacts of childhood socioeconomic disadvantage on later health outcomes are unclear [19]. For example, should more resources be allocated towards helping parents pursue higher education (e.g. the Single Mothers Academic Resource Team, SMART, in Oklahoma: http://www.smartok.org/, accessed June 28, 2017), or should there be more focus on ameliorating financial disadvantage (e.g. through transfer payments)? In this way, when operationalizations of cSES that combine multiple constructs are used in research, results do not provide guidance on which constructs should be the target of interventions to address socio-economic disparities.

**Fig 1:**
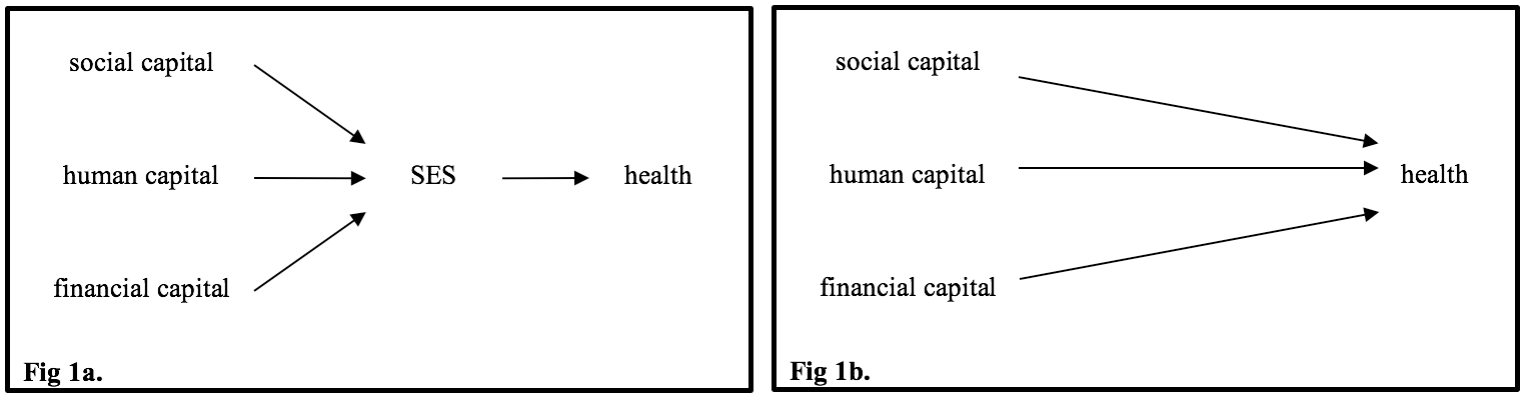
Options for modelling the relationship between SES and Health. There are conceptual and disciplinary differences in the functional form assumed to describe the relationship between SES and health. Some researchers posit that social capital, human capital, and financial capital have common effects (i.e. are mutually exchangeable), meaning an SES index is appropriate (Fig 1a). Other researchers posit that each form of capital has a distinct effect on health, and therefore each variable should be included in regression models separately (Fig 1b). Depending on theoretical orientation and the research question, one specification may be more appropriate than another. We validate measures of childhood social capital, childhood financial capital, and childhood human capital, which can be used independently or combined into a single cSES index; we note, however, that combining the measures into an index is likely a violation of the consistency assumption for causal inference [18,19].

In this paper, we apply an explicit conceptual framework to the measurement of cSES using variables available in the Health and Retirement Study (HRS, which is nationally representative of community-residing United States adults aged 50+ years), and use psychometric techniques to validate these measures, resulting in validated measures of childhood social capital, financial capital, and human capital; these measures can be used independently or combined into a single cSES index (Fig 1). We go on to compare the performance of the validated measures to other cSES operationalizations previously used in HRS research with respect to quality of predictions (adjusted R^2^ for predicting adult self-rated health and number of depressive symptoms [20]) and achievable sample size. Validation of cSES measures in HRS is important because the cohort has been widely used to test empirical questions, HRS has rich data on social conditions throughout the lifecourse, and HRS has numerous international sister-studies, offering the potential for international data harmonization. HRS, like many large, long-running, cohorts, has several conceptually related but not perfectly consistent measures of cSES, and includes more comprehensive, detailed assessments on selected subsamples or experimental modules embedded within the larger cohort. In theory, these subsample measures could substantially improve interpretation of the cruder measures available on the full sample. However, formal latent variable models to take advantage of the subsample cSES data have rarely been employed.

### 1.1 Theoretical perspectives on socio-economic status

We broadly follow the conceptual framework for cSES proposed by Entwisle and Astone [21], arguing that three types of capital are important for childhood development: financial capital, human capital, and social capital. A similar theoretical framework was proposed by Oakes and Rossi [15]. The Entwisle/Oakes frameworks draw on the conceptualization of social stratification developed by the sociologist James Coleman, who suggested that power differentials arise from inequalities between individuals with regard to their interests and control over scarce resources [22]. Such resources may take the form of (1) material and monetary goods (financial capital), (2) skills and capabilities (human capital), and (3) the strength and quality of social relationships (social capital). However, there are other ways to define these types of capital, see for example, Osterbacka et al., 2010, who posit time spent with children is a form of human capital [23], whereas the Entwisle/Oakes framework considers time spent with children an indicator of social capital. We additionally recognize that these constructs may be conceptually better described as measures of socioeconomic position since they incorporate both status and resource based indicators [12], but we adopt the terminology socioeconomic status because it appears to remain predominant in the literature in this area.

In this analysis, we define financial capital as income or wealth, and hence command over material resources such as shelter, food, clothing, etc. Human capital refers to the stock of knowledge and skills and, with respect to childhood development, is often operationalized as parental educational attainment. Social capital is considered the presence and quality of social connections, either within a family, or from a family to the outside world [15,21]; social capital as related to children is defined as quality and number of relationships with household adults. Financial, human, and social capital have each been linked with various health outcomes, including mortality [24,25] and self-rated health [26].

Starting with this framework, we make adjustments based on data availability and the specific concerns of health researchers. First, we treat human capital, financial capital, and social capital as conceptually distinct constructs, requiring separate measurement models. We consider financial capital and social capital scales (requiring a reflective measurement model [27]), which are conceptualized as a pool of measurements correlated because they share a common cause (the latent variable) [28]. We consider human capital an index because, while mother’s educational attainment and father’s educational attainment may not share a common cause, they may have a common effect, requiring a formative measurement model [27,28]. Although we create measures of human, financial, and social capital separately, these constructs will often be correlated.

Second, the Entwisle/Oakes framework suggests human, financial, and social capital have common effects, and can therefore be combined into a single childhood SES index (Fig 1a). Our approach also allows for the possibility that each form of capital has a distinct relationship with specific dimensions of health [29] (Fig 1b). In this analysis, we generate separate measures for each form of capital and provide guidance for creating a single cSES index, consistent with the approach adopted by Entwisle/Oakes.

### 1.2 Missing data and retrospective measurement of cSES

A primary challenge with retrospective assessments of cSES is missing data: many respondents do not know or cannot recall information about their childhood. Missing data may be particularly prevalent among older adults, who are vulnerable to cognitive decline [30]. Further, some questions commonly used to assess cSES are not appropriate for all respondents, or the missingness may be informative; for example, missing data on father’s education may indicate childhood family structure did not include a co-resident father [11]. A complete case approach would exclude these respondents, who may be the most socially disadvantaged. Even more sophisticated approaches to missing data, such as multiple imputation, would fail to appropriately incorporate this type of information if the missingness was informative.

We are able to address some missing data issues in this analysis by estimating dimensions of cSES using latent variable models whenever possible. Each latent variable is considered the cause of item responses, meaning all items in a scale have the same underlying cause of co-variation [28]. Scale scores can be imputed for individuals who are missing data on some, but not all, of the items that make up the scale. Through full information confirmatory factor analysis, we are able to impute scale scores for respondents who answered at least one of the items in the scale. This approach also allows for efficient and more complete use of data subsamples (several items used in the cSC and cFC scales came from experimental modules, which were sent to a random subsample of respondents). Second, we use iterative maximum likelihood estimation to impute parental education values for individuals who are missing this information. This option is often substantially more appealing than selectively deleting individuals with missing data on key variables, using a missing indicator, or mean imputation of missing information [31,32].

In this paper, we advance the literature in several ways. We apply a comprehensive theoretical framework for measuring childhood SES, as proposed by Entwisle/Oakes, in a data set with rich characterization of childhood social conditions and health information. We use robust methods to impute latent variable values in the presence of missing data. We employ psychometric tools to assess the internal consistency reliability, construct validity, and predictive validity of our scales. Finally, we compare these validated measures to comprehensive measures created by other HRS researchers [3,11,33] on quality of predictions (adjusted R^2^ in predicting adult self-rated health and number of depressive symptoms) and achieved sample size to demonstrate the comparative effectiveness of these measures in contrast to other comprehensive operationalizations.

## 2 Methods

Methods for this paper are presented in two sections; the first section (2.1) details how cSES scales were developed and validated, the second section (2.2) describes analyses comparing these validated measures to previous cSES indices created and used in HRS.

### 2.1 Development and validation of measures of childhood SES

#### 2.1.1 Sample

Data come from HRS, which began in 1992 as a nationally representative survey of non-institutionalized individuals born 1931–1941 [34]; in 1993, data were collected on a nationally representative sample of people aged 70 and older in the study of Assets and Health Dynamics of the Oldest Old (AHEAD), which included people born 1890–1923 [35]. In 1998, the two studies merged, and cohorts born 1924–1930 and 1942–1947 were added to form a nationally representative sample of individuals 50 years of age and older. To maintain a “steady state” population, new enrollments of people aged 50–56 are added every six years [36]. After enrollment, study members are re-interviewed approximately every 2 years.

**Fig 2:**
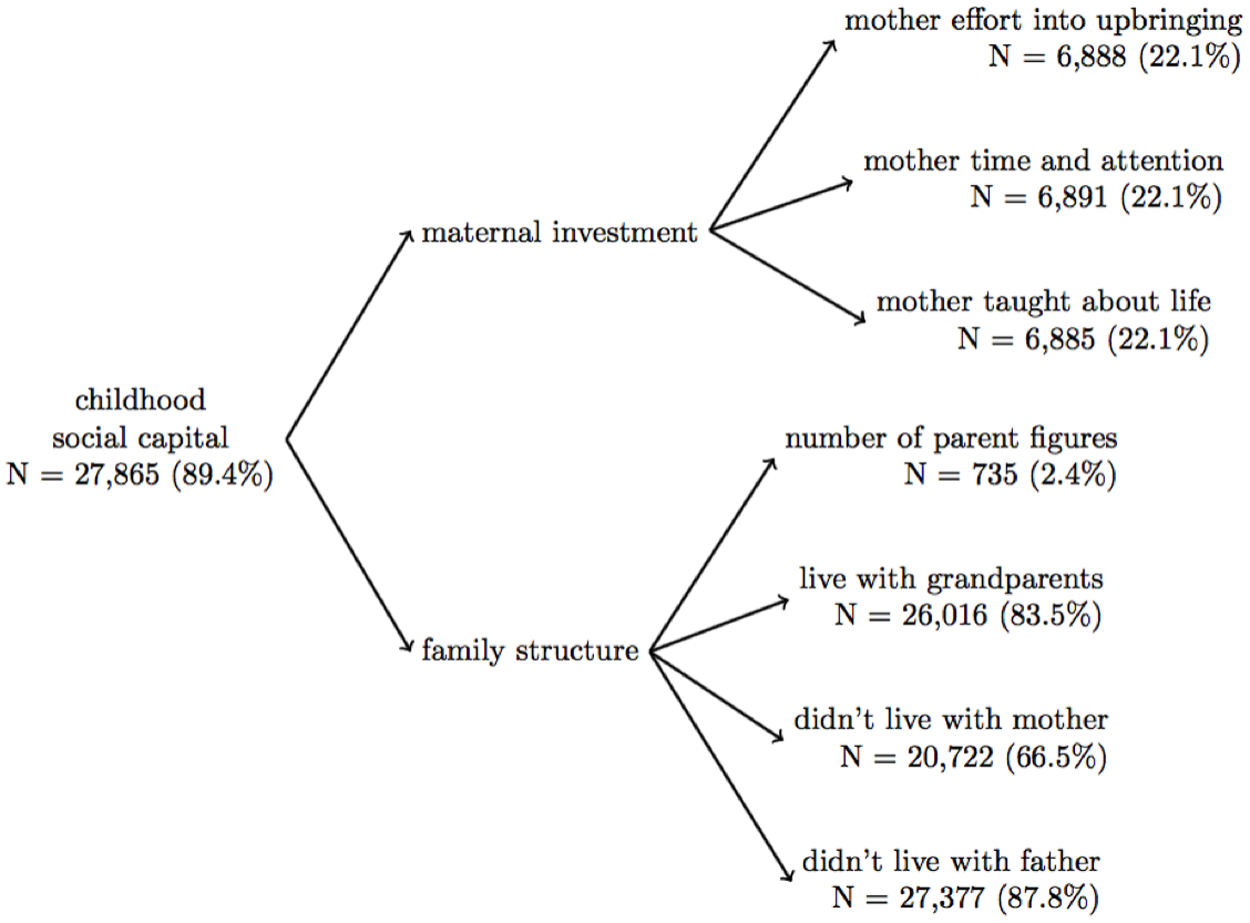
Factor structure for the social capital scale. We found that, as hypothesized, a two-factor solution best fit our data for the childhood social capital scale. Although we had limited data for some questions (i.e. number of parent figures was only available for 2.4% of the sample), through full-information confirmatory factor analysis, we were able to impute scale scores for 89.4% of our sample.

**Fig 3:**
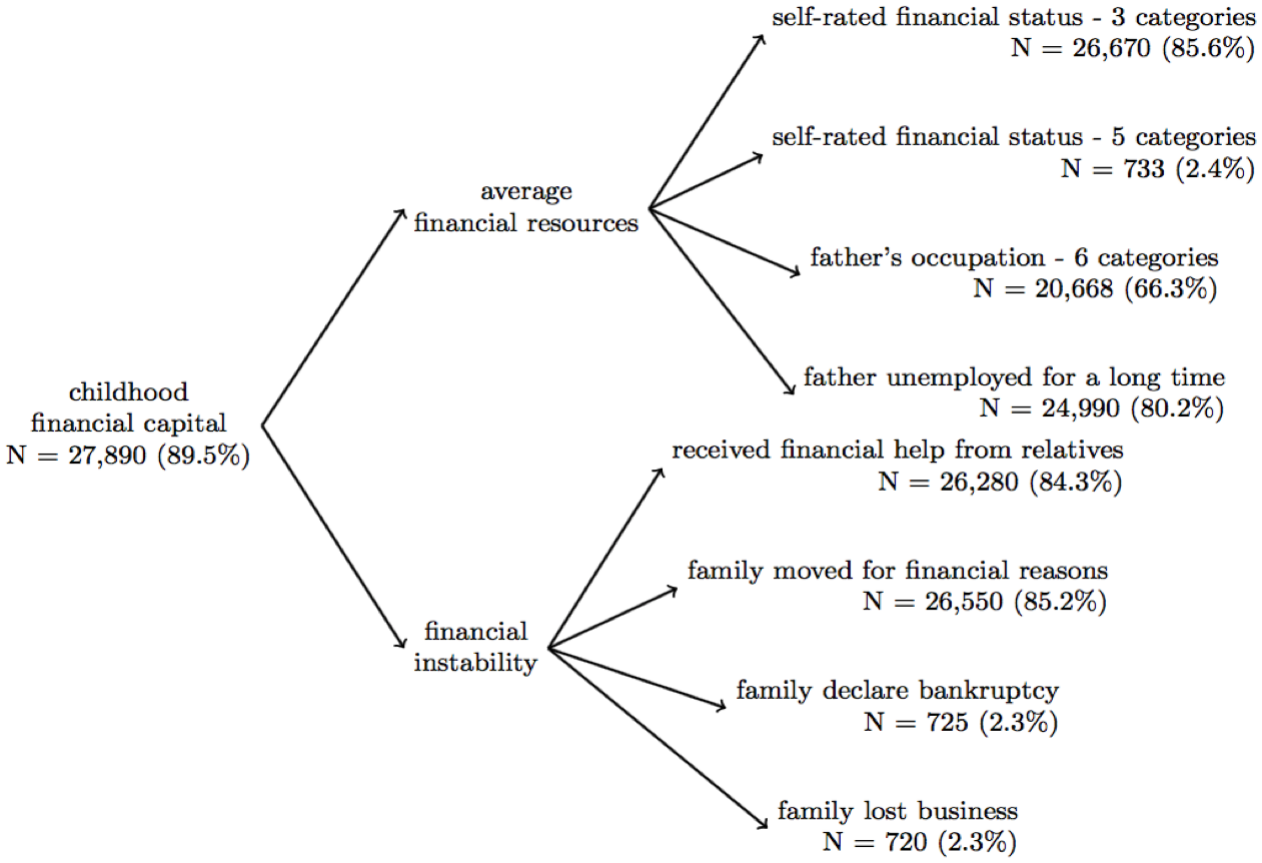
Factor structure for the financial capital scale. We found that a two-factor solution best fit our data for the childhood financial capital scale (we had hypothesized a one-factor solution, see S1 Fig for details). Although we had limited data for some questions (i.e. data on if the respondent’s family declared bankruptcy before at 16 was only available for 2.3% of the sample), through full-information confirmatory factor analysis, we were able to impute scale scores for 89.5% of our sample.

**Fig 4:**
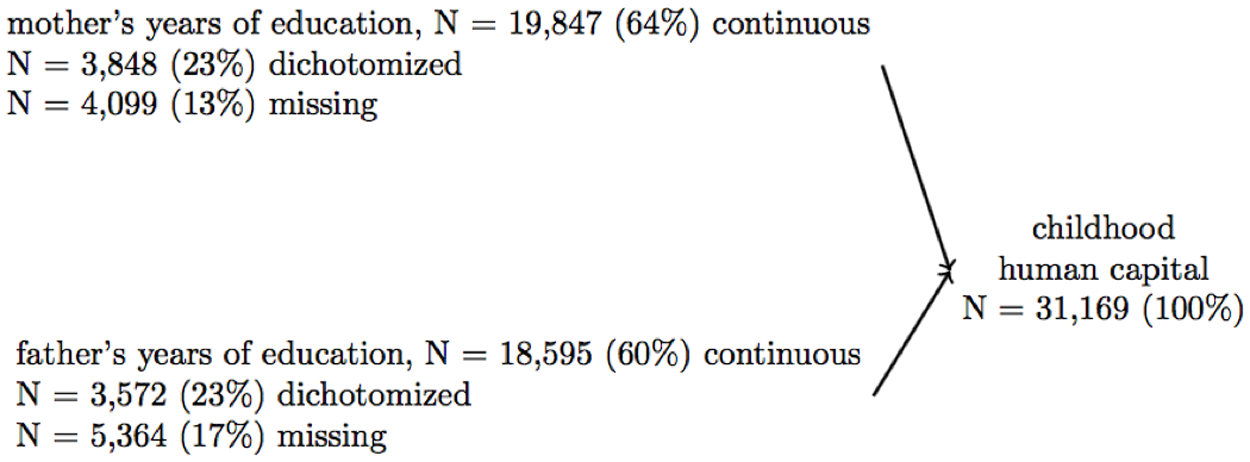
Structure of human capital index. For the childhood human capital index, data on parental education were recorded from 0–17 years for 64% of mothers and 60% of fathers, data were recorded dichotomized at 8 years for 23% of mothers and 23% of fathers, and data were missing for 13% of mothers and 17% of fathers. Through using expectation maximization (more details in S3 Table), we were able to impute continuous education information for 100% of the sample. We used expectation maximization rather than full information confirmatory factor analysis (which was used for the social and financial capital scales) because we conceptualized human capital as an index.

Self- and proxy- reported data on all participants who were interviewed on or before 2008 (N = 31,169) were used. We used many variables in this analysis that were first asked in experimental modules, and then incorporated into the main survey, meaning that the same question could have been asked to different respondents at different time points (see S1 Table for details). The number of observations differs across variables due to item non-response and the structure of the HRS questionnaire (i.e. some questions in experimental modules were asked to a subset of respondents, resulting in substantial missing data) (Figs 2, 3 and 4). All childhood measures were retrospectively reported and refer to the period when the respondent was <16 years; given that the youngest HRS participants are 50 years old, the childhood questions refer to a time period at least 34 years prior to when they were assessed.

#### 2.1.2 Childhood social capital scale

We hypothesized that the childhood social capital latent variable had two factors: a) maternal investment (assessed with three items) and b) family structure before age 16 (four items). The “maternal investment” factor included: 1) “How much effort did your mother put into watching over you and making sure you had a good upbringing?” (N = 6,888, 22.1%) 2) “How much did your mother teach you about life?” (N = 6,891, 22.1%) and 3) “How much time and attention did your mother give you when you needed it?” (N = 6,885, 20.1%); response options for these questions were: a lot, some, a little, and not at all. The “family structure” factor included 1) number of parent figures (operationalization described S2 Fig, N = 735, 2.4%), and if the respondent lived with 2) their mother (N = 20,722, 66.5%), 3) their father (27,377, 87.8%), 4) their grandparents (yes/ no) before age 16 (N = 26,016, 83.5%); the operationalization of living with mother and father are detailed in section 2.1.3, below (Fig 2; S1 and S2 Figs).

#### 2.1.3 Childhood financial capital scale

HRS assessed the following variables related to financial capital: 1) family moved for financial reasons before age 16 (yes/no, N = 26,550, 85.2%), 2) received financial help from relatives before age 16 (yes/no, N = 26,280, 84.3%), 3) family declared bankruptcy (yes/no, N = 725, 2.3%), 4) family lost business (yes/no, N = 720, 2.3%), 5) self-rated childhood SES, 3 categories (included in core questionnaire, response options: well off, about average, poor or varied; 276 (1.03%) who reported “it varied” recoded to “average”, N = 26,670, 85.6%), 6) self-rated childhood SES, 5 categories (included in experimental module, response options: very well off, above average, average, below average, very poor, N = 733, 2.4%), 4) the father’s occupation (six ordinal categories of a) executives & managers, b) professional specialty, c) sales & admin, d) protection services & armed forces, e) cleaning, building, food prep, and personal services, f) production, construction, and operation occupations; details: S2 Table, N = 20,668, 66.3%), 5) if the respondent’s father was unemployed for several months (yes, no, father never worked/always disabled, never lived with father/father was not alive; we recoded to ordinal categories of never worked/always disabled, lost job for several months, and not unemployed for several months, N = 24,990, 80.2%), and 6) if the respondent’s mother worked outside the house (all of the time, some of the time, not at all, never lived with mother/mother was not alive; we recoded to ordinal categories of mother worked all of the time, some of the time, not at all, N = 20,188, 64.8%). We considered the never lived with father (N = 2,459, 8.9%) and never lived with mother (N = 534, 2.6%) response options as markers of social capital rather than financial capital; therefore, this response option was coded as missing for the financial capital factor analysis (in order to impute a scale score), and included in the exploratory analysis for the social capital scale, detailed above (S1 Fig).

#### 2.1.4 Childhood human capital index

Human capital was operationalized as mother’s and father’s years of completed education (Fig 4). We conceptualized human capital as an index (not a scale) because mother’s and father’s education likely do not share a common cause of co-variation, although they are often correlated. Factor analysis is not appropriate for an index; in order to create a measure of human capital, we imputed values for individuals with missing or incomplete information (described below), z-scored both education variables, summed them to create a single human capital index, and then z-scored the index.

Due to inconsistent response formats across HRS survey waves, parent’s education was recorded as a continuous variable for most respondents (N = 19,847 mothers and 18,595 fathers), and dichotomized at ≥ 8 years for 7,013 mothers and 7,210 fathers; additionally, parental education data were missing for 4,095 mothers and 5,360 fathers. To create more complete measures of childhood human capital, we imputed continuous education values for mothers and fathers of respondents who had dichotomized or missing data using the iterative expectation maximization procedure (see S3 Table for details).

#### 2.1.5 Validation outcomes

To ascertain if the scales were measuring the intended constructs, we assessed the validity (detailed in section 2.1.7) through correlation with: measured adult height (data from 2008 and 2010), self-reported educational attainment (0–17 years), and retrospectively reported self-reported childhood health (excellent, very good, good, fair, poor), childhood learning problems (question text: “In grade school or high school, did you have a problem in learning the usual lessons, such that you regularly attended section classes, received special training sessions, or had to attend a different school for more than six months?”; response options: yes/no), and childhood drug or alcohol problems (question text: “Before you were 16 years old, did you have drug or alcohol problems?”; response options: yes/no).

#### 2.1.6 Factor analysis

We performed exploratory factor analysis to determine factor structure, and full-information confirmatory factor analysis to generate scale scores in the presence of missing data for both the cSC and cFC scales (factor analysis is not appropriate for the cHC measure because it is an index). We used geomin rotation and weighted least squares estimation because our data were categorical or ordered [37,38].

The number of factors was determined through eigenvalues and two measures of model fit: the root mean square error of approximation (RMSEA), which reflects how well the model fits the population’s covariance matrix (values < 0.07 reflect better fit [39]), and the comparative fit index (CFI), which compares the sample covariate matrix with a null model (values ≥ 0.95 reflect better fit [40]). Variables with factor loadings above 0.3 were retained in the scales. After the number of factors was determined, we used full-information weighted least squares confirmatory factor analysis to generate scores for the cSC and cFC scales for individuals with missing data on some scale items; individuals missing data on all scale items were excluded.

#### 2.1.7 Psychometric testing

Internal consistency reliability [28] was assessed for the cSC and cFC scales with Cronbach’s alpha. Construct validity, or the extent to which the measure correlates with theoretically relevant constructs [28], was assessed for cSC, cFC, and cHC through correlations with variables that reflect the respondent’s childhood experience, before age 16. We hypothesized that the cSC, cFC, and cHC scales would be negatively correlated with childhood learning problems (i.e. more capital correlated with fewer learning problems [41]), childhood drug and alcohol problems, as a proxy for contact with police [42], and childhood health (i.e. more capital correlated with better health [43,44]).

Predictive validity, or the ability of a scale to predict a future event [28], was assessed through correlation with variables that reflect health and social outcomes in adulthood. We hypothesized that the cSC, cFC, and cHC scales would be positively correlated with the respondent’s educational attainment (i.e. more capital would predict more education [45,46]), and measured height in 2008 or 2010 [47,48]. An additional check of predictive validity is presented in the comparison analysis described in section 2.2 below, as both outcomes (self-rated health and number of depressive symptoms) are strongly patterned by socio-economic status [49]; we hypothesized that more capital would be predict better self-rated health in adulthood, and fewer depressive symptoms in adulthood.

### 2.2 Comparison of validated measures to other comprehensive cSES operationalizations

#### 2.2.1 Sample

**Fig 5:**
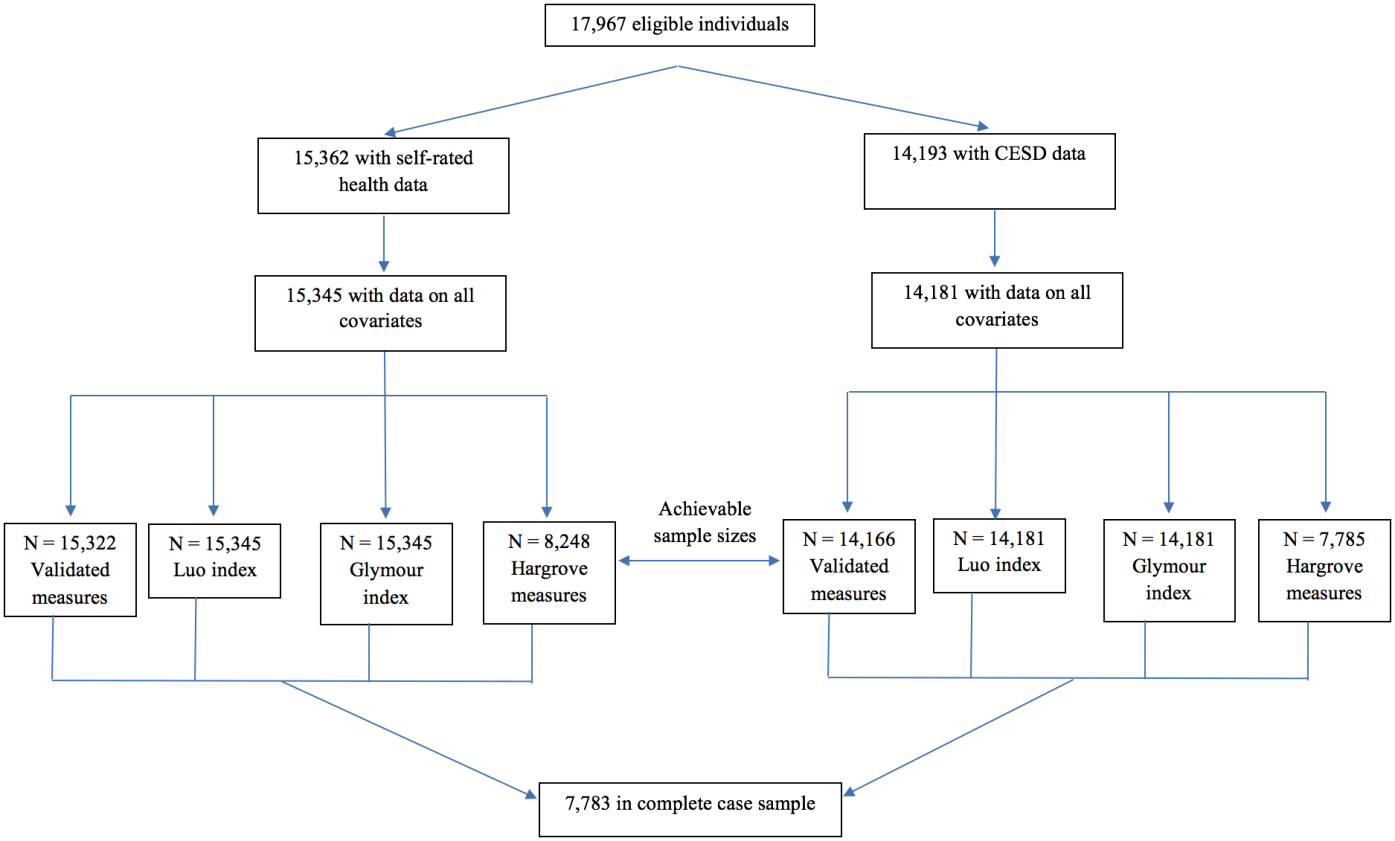
Flowchart of individuals included in the achievable sample size and complete case analyses.

In addition to creating and validating measures of childhood social circumstances, we compared our measures to previously developed measures of cSES in HRS data created by Luo [3], Glymour [11], and Hargrove [33] in two ways. First, we contrasted the proportion of variance explained by the alternative cSES measures for two adult health outcomes (self-rated health, and number of depressive symptoms, assessed with a modified Center for Epidemiologic Studies Depression Scale [50]). For these comparisons, we used a complete case analysis (N = 7,733) so identical sample sizes were available for each cSES analysis [20] (see Fig 5 for exclusions). In a second comparison analysis, we contrast the number of observations retained in analysis using our measures (N = 15,345) to the Luo (N = 15,345), Glymour (N = 15,345), and Hargrove (N = 8,248) comparison specifications of cSES (Fig 5).

#### 2.2.2 Creation of a cSES index from the validated measures

Details on how the measured created in this analysis were created are described in section 2.1, above. In a subset of the analyses a cSES index was created by combining the cSC, cFC, and cHC scales. All the measures were z-scored and averaged; if an individual was missing data on one or more component variables, the index was created by averaging the other variables. The final index was z-scored again for interpretability.

#### 2.2.3 Comparison cSES measures used in HRS data

Each of the three previously published cSES measures created in HRS data included only human and financial capital as components of cSES; none considered social capital part of cSES. Luo included four variables: mother ≥ 8 years of education, father ≥ 8 years of education, whether the father had a white-collar job, and whether respondent retrospectively described the family was financially “pretty well off”, “about average”, or “poor” (“it varied” was coded as missing and the cSES index was derived from the other variables (Ye Luo, personal communication)). Individuals with missing data on parent’s education or father’s occupation were considered low cSES. These variables were combined by standardizing, then averaging to create a continuous index [3].

Similarly, Glymour included mother’s and father’s years of education, and father’s occupation. These variables were combined into an index as follows: one point was given for known mother’s and father’s education < 8 years, and father’s manual occupation; unknown information did not necessarily indicate low cSES. The three items were averaged so the low childhood SES index ranged from 0 (best) to 1 (worst) in the original analysis, however, to facilitate comparison across measures, we reverse coded the index so 0 represented the lowest cSES and 1 represented the highest [11].

The Hargrove study included the following dichotomous indicators: mother’s education ≥ 12 years, father’s education ≥ 12 years (we believe Hargrove did not include the AHEAD cohort where parent’s education was dichotomized at 8), moving for financial reasons, self-rated cSES of poor, and father’s white collar occupation [33]. The Luo and Glymour measures of cSES each had a single variable and do not automatically exclude those with missing data (both researchers performed sensitivity analyses to ensure inclusion of individuals with missing data did not substantially change results); the Hargrove measure used five variables and a complete case approach.

#### 2.2.4 Outcomes

To compare the predictive ability of our measures with previously developed indexes of cSES in HRS, we examined two outcomes previously established to be strongly patterned by lifecourse SES: depressive symptoms and self-rated health [49], both measured in 2010. Self-rated health had five response options (excellent, very good, good, fair, and poor). Depressive symptoms over the previous week were assessed with a modified 8-item Center for Epidemiologic Studies Depression (CESD) scale summing yes/no responses to 6 “negative” items (feeling sad and depressed, everything is an effort, sleep is restless, feeling alone, and not able to get going) and two reverse-coded “positive” items (felt happy and enjoyed life); this scale is reliable among HRS participants (Cronbach’s alpha = 0.78) [51], and higher CESD scores indicated more depressive symptoms. The modified scale ranges from 0 to 8 and correlates with the original, 20-item scale [52].

#### 2.2.5 Covariates

All regression analyses adjusted for: age (linear and quadratic terms), gender, race/ethnicity (Non-Hispanic White (ref), Non-Hispanic Black, Hispanic, and Other Race), and birthplace indicators (north east (ref), south, mid-Atlantic, east north central, west north central, mountain, Pacific, or abroad).

#### 2.2.6 Analysis

Linear regression was used to compare performance of our measures to the other multi-component measures of cSES. In one analysis (complete case), we compared the amount of variability explained, assessed with the adjusted R^2^, when the number of observations was constant across the models (N = 7,783); prior work argues R^2^ can be used as a goodness of fit statistic and a way to assess model specification “when comparing two equations with different explanatory variables and identical dependent variables” [20]. In a second analysis (achievable N), we compared the number of observations retained for analysis with our measures to the Luo, Glymour, and Hargrove specifications.

Three specifications of our measures were evaluated. Model 1 included a cSES index comprised of our measures. Model 2 included the cSC, cFC, and cHC measures separately. Model 3 includes the components for all measures: maternal investment, family structure (factors for cSC), average financial resources, financial instability (factors for cFC), and mother’s and father’s education (components of cHC). All data cleaning and analyses were performed in SAS, version 9.3, except the factor analyses, which used Mplus, version 7. The code for these analyses is available on GitHub (https://github.com/anushavable/Validated-cSES-measures-in-HRS), and the measures we developed are available for download from the HRS data portal (https://hrs.isr.umich.edu/data-products/access-to-public-data).

## 3 Results

### 3.1 Validation of the childhood SES scales

#### 3.1.1 Childhood social capital

The cSC scale included two factors, maternal investment (3 items) and family structure (4 items), RMSEA = 0.009, CFI = 1.000 (Fig 2 and S4 Table), which were summed to create the cSC scale (S3 Fig). The final cSC scale was estimated for 27,865 respondents (89.4%); the cSC scale demonstrated acceptable internal consistency reliability (Cronbach’s alpha = 0.63), higher than the acceptable range of 0.5–0.6 for early research [53] (Table 1 and S5 Table), and good validity (correlation with childhood learning problems β = -0.11, p < 0.0001; childhood self-rated health β = -0.03, p < 0.0001, childhood drug and alcohol problems β = -0.16, p = 0.0086, educational attainment β = 0.01, p < 0.0001; and measured height β = 0.01, p < 0.0001; adult self-rated health β = -0.05, p < 0.0001, number of depressive symptoms β = -0.12, p < 0.0001) (Tables 2 and 3, Model 2).

**Table 1:** Internal consistency reliability of the childhood social capital, and financial capital scales.

Scale	N	Standardized Cronbach’s alpha
Childhood social capital	226	0.63
Maternal investment	6871	0.89
Family structure	595	0.52
Childhood financial capital	657	0.63
Average financial resources	664	0.56
Financial instability	718	0.74

Reliability is assessed among individuals who have data on all scale items; many of the questions included in the social and financial capital scales were included in experimental modules, resulting in relatively small Ns for the relatability calculation. It is not appropriate to calculate the reliability of an index, so cHC is not included in this table.

**Table 2:** Linear regression models evaluating relationships between childhood SES domains and theoretical correlates.

		Childhood social capital			Childhood financial capital			Childhood human capital		
	Predictors	N	β	p-value	N	β	p-value	N	β	p-value
	Childhood financial capital	27,690	0.07	<.0001						
	Childhood human capital	27,865	0.01	0.0241	27,890	0.21	<.0001			
Construct validity	Childhood drug / alcohol problems	13,370	-0.16	0.009	13,353	-0.34	<.0001	13,370	0.13	0.235
	Childhood learning problems	16,626	-0.11	<.0001	16,606	-0.21	<.0001	16,626	-0.05	0.292
	Childhood self-rated health	26,663	-0.03	<.0001	26,682	-0.10	<.0001	26,714	-0.17	<.0001
Predictive validity	Educational attainment	27,804	0.01	<.0001	27,829	0.04	<.0001	30,677	0.16	<.0001
	Measured height	12,844	0.01	<.0001	12,837	0.002	0.19	12,844	0.04	<.0001

All betas are linear regression coefficients; the row variables predicted the column variables. Childhood social, financial, and human capital, educational attainment, and measured height are coded so higher numbers reflect better properties; childhood drug / alcohol problems, learning problems, and self-rated health is coded so lower numbers reflect better properties. Childhood drug / alcohol problems, learning problems, and self-rated health were used to assess construct validity, while educational attainment and measured height were used to assess predictive validity.

Our finding that childhood financial capital is not associated with adult height is contrary to the literature on cSES and adult height. We conducted supplemental analyses to understand these discrepant findings and concluded that the observed differences are likely due to differences in the way cSES is operationalized across studies. Our results suggest that the relationship between cSES and adult height is primarily through parental education, however similar analyses should be conducted in different samples to confirm or refute these findings.

**Table 3:** Complete case comparison of validated measures with other comprehensive measures (N = 7,783) predicting self-rated health and number of depressive symptoms.

		Self-rated health				Number of depressive symptoms			
		Beta	(95%CI)	p	Adj. R^2^	Beta	(95%CI)	p	Adj. R^2^
**Validated Measures**									
Model 1	cSES index	-0.18	(-0.20,-0.15)	<.0001	0.070	-0.25	(-0.29,-0.21)	<.0001	0.049
Model 2	cSC	-0.05	(-0.07,-0.03)	<.0001	0.070	-0.12	(-0.16,-0.09)	<.0001	0.050
	cFC	-0.06	(-0.09,-0.04)	<.0001		-0.11	(-0.15,-0.07)	<.0001	
	cHC	-0.15	(-0.17,-0.12)	<.0001		-0.15	(-0.20,-0.10)	<.0001	
Model 3	Maternal investment	-0.15	(-0.23,-0.07)	0.0002	0.071	-0.32	(-0.46,-0.18)	<.0001	0.050
	Family structure	0.11	(-0.16,0.37)	0.430		0.07	(-0.40,0.54)	0.782	
	Average financial resources	-0.14	(-0.43,0.14)	0.325		0.38	(-0.12,0.89)	0.137	
	Financial instability	0.06	(-0.10,0.21)	0.471		0.43	(0.15,0.70)	0.002	
	Mother’s education	-0.10	(-0.13,-0.07)	<.0001		-0.10	(-0.16,-0.04)	0.0004	
	Father’s education	-0.06	(-0.09,-0.03)	0.0003		-0.07	(-0.13,-0.02)	0.018	
**Comparison Measures**									
Luo Index		-0.14	(-0.16,-0.12)	<.0001	0.060	-0.15	(-0.19,-0.10)	<.0001	0.040
Glymour Index		-0.45	(-0.52,-0.37)	<.0001	0.060	-0.46	(-0.59,-0.32)	<.0001	0.039
Hargrove measures	Mother’s education ≥ 12	-0.17	(-0.22,-0.11)	<.0001	0.064	-0.15	(-0.25,-0.06)	0.002	0.041
	Father’s education ≥ 12	-0.09	(-0.14,-0.03)	0.002		-0.12	(-0.22,-0.02)	0.020	
	Self-rated poor SES	0.09	(0.03,0.14)	0.002		0.12	(0.02,0.22)	0.014	
	Moved for financial reasons	0.11	(0.05,0.17)	0.001		0.23	(0.11,0.34)	<0.0001	
	Father occupation	-0.10	(-0.17,-0.04)	0.002		0.02	(-0.10,0.13)	0.80	

Self-related health and CESD score are coded so lower numbers reflect better health.

All of the validated measures, are coded so that higher number reflect more capital; financial instability, is coded so higher numbers reflect more financial instability.

All models are adjusted for age (linear and quadratic terms), race / ethnicity, gender, and birthplace. The cSES index, cHC, cFC, cSC, Luo index, as well as, mother’s years of education, and father’s years of education in Model 3 were all z-scored so a one-unit change represents a change of 1-standard deviation.

Exclusion of the socially vulnerable in the complete case analysis induced a (non-statistically significant, p = 0.14) spurious relationship between average financial resources and number of depressive symptoms such that more financial resources predicts more depressive symptoms, which contradicts past literature. In the achievable N analysis (Table 4) the socially vulnerable are included, pushing this relationship towards the null (p = 0.55).

The change in variability explained from 0.060 (Luo and Glymour indices) to 0.070 (the cSES index, Model 1) for self-rated health represents a 17.7% increase in variability explained; such an increase in variance explained would concomitantly improve statistical power or reduce necessary sample size to detect an association. To contextualize this change in variability, a one-percentage point increase in explained variability (i.e. 0.060 to 0.070) is more than double the variability explained by age (linear and quadratic terms) and gender combined (R^2^ = 0.0046). Simulation results (with 10,000 repetitions) reveal that, given two measures that explain 7% of the variability in the outcome, a difference in R^2^ as big a 0.01 occurs 2.6% of the time when N = 7,783, indicating that this difference is statistically significant.

#### 3.1.2 Childhood financial capital

The cFC scale included two factors, average financial resources (4 items) and financial instability (4 items), RMSEA = 0.037, CFI = 0.954 (Fig 2 and S4 Table), which were summed to create the cFC scale (S3 Fig). The cFC scale was estimated for 27,890 respondents (89.5%), and demonstrated acceptable internal consistency reliability (Cronbach’s α = 0.61) (Table 1 and S6 Table), and validity (correlation with childhood learning problems β = -0.21, p < 0.0001; childhood self-rated health β = -0.10, p < 0.0001; childhood drug and alcohol problems β = -0.34, p < 0.0001; educational attainment β = 0.04, p < 0.0001; measured adult height β = 0.002, p = 0.19; adult self-rated health β = -0.06, p < 0.0001; number of depressive symptoms β = -0.14, p < 0.0001) (Tables 2 and 3, Model 2).

#### 3.1.3 Childhood human capital

The childhood human capital (cHC) measure was estimated for 31,169 respondents (100%), and included variables on mother’s and father’s years of education (Fig 4 and S3 Fig). The cHC demonstrated acceptable validity (correlation with childhood learning problems = -0.02, p = 0.12, childhood self-rated health β = -0.16, p<0.0001; childhood drug and alcohol problems β = 0.09, p = 0.41; educational attainment β = 0.15, p < 0.0001; measured adult height β = 0.04, p < 00001; adult self-rated health β = -0.15, p < 0.0001; number of depressive symptoms β = -0.15, p < 0.0001) (Tables 2 and 3, Model 2).

### 3.2 Comparison of validated measures to previous cSES operationalizations

#### 3.2.1 Complete case analyses

There were 7,783 individuals in the complete case analysis (Fig 5). The cSES index explained 7.0% of the variability in self-rated health (β = -0.18; 95%CI: -0.20, -0.15; p <0.0001), and 4.9% of the variability in number of depressive symptoms (β = -0.25; 95%CI: -0.29, -0.21; p < 0.0001) (Table 3, Model 1). The individual cSES measures predicted both self-rated health (7.0% of variability explained) and number of depressive symptoms (5.0% of variability explained) (Table 3, Model 2). Inclusion of component measures did not change the proportion of variability explained (Table 3, Model 3).

The Luo, Glymour, and Hargrove measures of cSES significantly predicted self-rated health and number of depressive symptoms, though the models explained less of the variability in the outcome than the validated measures. The Luo cSES and Glymour indices explained 6.0% of the variability in adult self-rated health, while the Hargrove measures explained 6.4%. For number of depressive symptoms, the Luo index explained 4.0% of the variability, the Glymour measure explained 3.9%, and the Hargrove measure explained 4.1% of the variability.

#### 3.2.2 Achievable N analyses

Using all available cases increased the sample substantially: the validated cSES index had a sample size of 15,345 for self-rated health and 14,181 for number of depressive symptoms. The Luo and Glymour indices both had sample sizes of 15,345 for self-rated health and 14,181 for number of depressive symptoms; the Hargrove model had a sample size of 8,248 for self-rated health and 7,785 for number of depressive symptoms (Fig 5 and Table 4). Although coefficients were generally similar when estimated in the smaller complete case data set (Table 3), estimates were much more precise in the larger available-case sample (Table 4). An exception was the relationship between average financial resources and for number of depressive symptoms, which appeared borderline significant in the complete case analysis (β = 0.38; 95%CI: -0.12, 0.89; p = 0.137, Table 3, Model 3), but showed no relationship in the achievable N analysis (β = -0.13; 95%CI: -0.26, 0.53, p = 0.51, Table 4, Model 3).

**Table 4:** Comparison of validated measures with other comprehensive measures on self-rated health and number of depressive symptoms, using all available cases.

		Self-rated health				Number of depressive symptoms			
		N	Beta	(95%CI)	p	N	Beta	(95%CI)	p
**Validated Measures**									
Model 1	Childhood SES Index	15,345	-0.19	(-0.20,-0.17)	<.0001	14,181	-0.32	(-0.35,-0.29)	<.0001
Model 2	cSC	15,322	-0.06	(-0.07,-0.04)	<.0001	14,166	-0.16	(-0.19,-0.13)	<.0001
	cFC		-0.06	(-0.08,-0.05)	<.0001		-0.14	(-0.17,-0.10)	<.0001
	cHC		-0.16	(-0.18,-0.14)	<.0001		-0.19	(-0.23,-0.15)	<.0001
Model 3	Maternal investment	15,322	-0.13	(-0.19,-0.08)	<.0001	14,166	-0.36	(-0.46,-0.26)	<.0001
	Family structure		0.004	(-0.14,0.15)	0.962		-0.02	(-0.30,0.25)	0.870
	Average financial resources		-0.11	(-0.32,0.10)	0.291		0.12	(-0.27,0.52)	0.545
	Financial instability		0.07	(-0.04,0.18)	0.248		0.34	(0.13,0.55)	0.002
	Mother’s education		-0.08	(-0.11,-0.06)	<.0001		-0.12	(-0.16,-0.07)	<.0001
	Father’s education		-0.08	(-0.11,-0.06)	<.0001		-0.09	(-0.14,-0.05)	<.0001
**Comparison Measures**									
Luo Index		15,345	-0.17	(-0.19,-0.15)	<.0001	14,181	-0.25	(-0.28,-0.22)	<.0001
Glymour Index		15,345	-0.30	(-0.36,-0.24)	<.0001	14,181	-0.26	(-0.37,-0.15)	<.0001
Hargrove measures	Mother’s education ≥ 12	8,248	-0.17	(-0.22,-0.12)	<.0001	7,785	-0.15	(-0.25,-0.05)	0.002
	Father’s education ≥ 12		-0.11	(-0.16,-0.05)	0.0002		-0.12	(-0.22,-0.02)	0.020
	Self-rated poor SES		0.06	(0.01,0.11)	0.026		0.12	(0.02,0.22)	0.014
	Moved for financial reasons		0.11	(0.04,0.17)	0.001		0.23	(0.11,0.34)	<.0001
	Father occupation		-0.10	(-0.16,-0.04)	0.002		0.01	(-0.10,0.13)	0.812

Self-related health and CESD score are coded so lower numbers reflect better health.

All of the validated measures are coded so that higher number reflect more capital; financial instability, is coded so higher numbers reflect more financial instability.

All models are adjusted for age (linear and quadratic terms), race / ethnicity, gender, and birthplace. The cSES index, cHC, cFC, cSC, Luo index, as well as, mother’s years of education, father’s years of education in Model 3 were all z-scored so a one-unit change represents a change of 1-standard deviation.

## 4 Discussion

Using HRS, a nationally representative cohort that has been particularly influential in lifecourse and aging research, we developed and validated a theoretically motivated index of cSES. Our measures demonstrated acceptable to good internal consistency reliability, construct validity, and predictive validity. Our validated measures outperformed previous multi-item indexes proposed by Luo et al., Glymour et al., and Hargrove et al. with respect to proportion of variability explained for adult self-rated health and depressive symptoms in these data. By using a latent variable model, the validated measures allow analysts to retain respondents with partial missingness, increasing available observations compared to a complete case approach. This increase in sample size improved statistical power and reduced bias in the relationship between average financial resources and number of depressive symptoms by including the socially vulnerable who were otherwise excluded.

Despite the popularity of the HRS cohort for lifecourse research, no consensus has emerged regarding the optimal operationalization of cSES. Various studies select different items for assessment of cSES and use alternative algorithms to combine the selected items. This inconsistency is exacerbated by changes in the HRS questionnaire over time and the availability of enhanced measures on subsamples of the cohort. The cSES index developed here offers several advantages, including subscales related to theoretically relevant dimensions of cSES, efficient use of incomplete data, and modestly improved prediction of adult health.

### 4.1 Missing data approach benefits

Our approach to handling missing data had two benefits. First, the effective sample size increased; more data points provide more power, which may be particularly important for subgroup analyses. Second, our measures reduce bias in point estimates by including the most socially disadvantaged individuals who are excluded from complete case analyses. Hargrove & Brown excluded individuals with missing data, implicitly relying on a missing completely at random (MCAR) assumption, that missingness is not patterned by cSES [31]. However, analysis of HRS data suggests that data on a parent’s education is missing for respondents who did not live with that parent [11], indicating the missingness is patterned by household structure, likely impacting childhood financial and human capital. Our analysis shows that individuals excluded when cSES is based on the Hargrove model are indeed more socially vulnerable than included individuals (S7 Table).

When missing data are patterned, the missingness should be modeled in order to produce unbiased point estimates [31]. In this analysis, we imputed scale scores for individuals with missing data, substantially reducing missing data among the most socially vulnerable, and reducing bias in point estimates. The point estimate for the relationship between average financial resources and number of depressive symptoms was positive in complete case analysis (and borderline statistically significant, p = 0.14) indicating that *more* financial resources in childhood predicted *more* depressive symptoms (Table 3), which is the inverse of the relationship found in past literature (i.e. prior work suggests those with lower SES have more depressive symptoms [54]). In the achievable N analysis, on the other hand, the point estimate is much smaller, and the p-value is quite large (p = 0.51), indicating that the relationship between average financial resources and number of depressive symptoms is null (Table 4, Model 3). In subsequent analyses, we discovered that socially vulnerable individuals excluded from the complete case analysis also had more depressive symptoms (S4 Fig and S8 Table), pushing the relationship between average financial resources and number of depressive symptoms from a positive relationship in the complete case analysis (contrary to the literature) to a null relationship in the achievable N analysis, which is more consistent with the literature. These results suggest that excluding socially vulnerable individuals in the complete case analysis induced a (non-statistically significant) relationship between average financial resources and number of depressive symptoms, which disappeared in the larger achievable N sample, where the socially vulnerable were not excluded.

While there are still missing data with the validated measures, our imputation approach generated scale scores among socially vulnerable groups excluded in complete case analyses, decreasing bias in point estimates.

### 4.2 The consistency assumption, understanding health, and informing interventions

Some benefits of operationalizations of cSES that enable estimation of different point estimates for different component measures (i.e. Table 4, Models 2 & 3) include a more nuanced understanding of these relationships and the ability to inform future interventions. For example, cFC exerts a strong influence on number of depressive symptoms (Table 4, Model 2), indicating that childhood financial resources are very important for adult mental health. However, because we were able to separate childhood financial capital into two factors, average financial resources and financial instability, we found that childhood financial instability really matters for adult mental health, while our results suggest average financial resources in childhood has no relationship with adult mental health (Table 4, Model 3).

Additionally, a well-defined treatment is one of the assumptions of causal inference [18]; meeting this assumption and can help researchers interpret their findings to inform future interventions [19], provided other assumptions for casual inference are also fulfilled [55]. As applied to the example of the factor scores for cFC, our results suggest that programs that mitigate financial shocks for families with children, such as reducing medical bankruptcy [56], more generous unemployment benefits, or increasing the time frame for Temporary Assistant for Needy Families [57], may help reduce the population prevalence of depression among older adults. This is particularly important because recent research suggests that childhood financial conditions have a direct effect on adult health (while the effect of parental education is mediated through own education)[58], suggesting the deleterious effects of financial instability in childhood may not be offset by socio-economic gains later in life.

### 4.3 Limitations

Although the validated cSES measures are an improvement, there are many limitations to developing scales using existing data. While HRS includes many important questions on early life social circumstances, notable gaps in HRS topics include, but are not limited to: a) more complete assessment of maternal investment, b) measures of the social investment of the respondent’s father, and c) assessments of important skill sets such as language (i.e. speaking Spanish), music skills, or trade skills (i.e. carpentry, plumbing, electrical, etc.) which are not necessarily correlated with years of formal education. These measures additionally offer little insight why these measures of capital may be low, and important explanatory factors, such as whether mothers invested limited time in the child due to competing demands from other family members or paid labor, etc. were not assessed. It is also possible that other social capital measures than the ones available in HRS, such as neighborhood deprivation or civic engagement, are also relevant dimensions for health. Reliability for the cSC and cFC scales were relative low (around 0.6); such a low reliability may bias effects towards the null and lead to residual confounding if analyses do not account for measurement error [59]. The standard errors for the scales in Table 3 should be interpreted with caution because our analyses did not account for the two-stage estimation strategy. No gold standards for these dimensions of cSES are available to assess criterion validity; we relied on imperfect and retrospectively reported indicators to assess construct and predictive validity. This analysis used subjective SES measures (i.e. self-reported cSES), however, objective measures (i.e. number of bedrooms in the house) are likely measured with more precision [60–62]. Several of the questions retained in the cSC and cFC scale were asked only to a randomly selected subset of respondents (N < 7,000) in experimental modules; we were able to impute scale scores for many individuals who were not asked these questions with our latent variable approach, however the small sample size likely reduced precision (Fig 2 and S3 Fig). Additionally, the response options “didn’t live with mother” and “didn’t live with father” were derived from one of several response options to questions on parental employment; to reduce ambiguity on whether the respondent lived with either of their parents this question should be asked directly. Lastly, cSES can vary over the respondent’s childhood; we were not able to capture time variation in cSES in these analyses.

All of the measures used in this analysis are retrospectively self-reported, which may introduce measurement error and bias. Sources of measurement error include the time interval and degree of detail remembered [63], and differential recall by adult social class [60,64], or misreporting due to poor mental, physical, or cognitive health at the time of data collection. Prior work has found acceptable concordance of retrospectively reported social class with maternity records and prior self-report [65], historical records [62], and birth and census records [66], though some researchers find adults retrospectively report more favorable cSES than was recorded during childhood [65]. Additionally, research on concordance of siblings’ self-report finds high concordance for the head of household’s occupation [67] and receipt of welfare benefits but concordance was lower when at least one sibling had a high school education or less [60]. We are not able to quantify the amount of measurement error or degree of resulting bias in these measures. While these sources of bias are a cause for concern, alternative approaches of collecting information on cSES among adults, such as obtaining these data from historical records [62], birth certificates, or census data [66], may be prohibitively time-consuming, expensive, may only cover a subset of the relevant content domains, and are relatively rare [7,8,68].

A final limitation of this work is that the variables used to create our scales may not be relevant for today’s children, and, therefore, we do not necessarily recommend inclusion of these questions in surveys in other historical or cultural settings. For example, mother’s employment status for this cohort of older adults may indicate low childhood financial capital, whereas it is normative for both parents to work today, and a working mother may indicate high childhood financial capital. Additionally, growing up with one’s grandparents may indicate absent biological parents in the US, suggesting low childhood social capital, whereas intergenerational households are normative in other countries and may not reflect childhood social capital. While we advanced the literature by applying this framework to a dataset with rich characterization of lifecourse social conditions, we believe that the measurement of cSES could be substantially improved through new question development and primary data collection.

### 4.4 Conclusions

This work builds and improves upon previous indicators of childhood SES in several ways. Substantively, this work is an advance in developing six distinct constructs (maternal investment and family structure as measures of cSC, average financial resources and financial instability as measures of cFC, and more complete measures of mother’s and father’s education as measures of cHC), which allows for many flexible specifications (i.e. examining additive or interactive effects, or combining the measures into a single cSES index; we note, however, that combining the measures into an index is likely a violation of the consistency assumption for causal inference [18,19]). These measures may also help refine our understanding of the relationship between cSES and health outcomes. For example, our analysis reveals that childhood financial instability and childhood maternal investment have large and independent effects on number of depressive symptoms in adulthood; additionally, these effects are larger than other commonly used markers of cSES, such as average financial resources or parental education. As far as we know, the size of these childhood exposures on number of depressive symptoms among older adults are new findings that we were able to uncover because we used psychometric approaches to measure cSES.

Implementing these scales in future HRS research will advance the field methodologically by helping to improve consistency in the measurement of cSES, which can inform future intervention and facilitate meta-analyses. Further, as we demonstrated with the point estimate for average financial resources, using these measures can reduce bias in point estimates compared to complete case approaches which may exclude socially vulnerable groups.

However, this work also highlights several remaining gaps in the measurement of cSES. Theoretical developments on lifecourse SES and older adult health have largely outpaced the quality of data available to test those theories. Valuable data linkages could be made with a little more information; for example, if HRS collected information on female respondent’s maiden name, the household of residence could be established through linkages with census data. Similarly, data on the respondent’s elementary and high schools could be linked to data on graduation rates and school quality to assess whether differences in educational institution explain heterogeneities in older adult health and well-being.

Outside of HRS, researchers should adopt this conceptually driven approach by creating cSES measures in other datasets and though primary data collection. Wider adoption of measurement theory will improve consistency across datasets, and, as demonstrated in our analysis, may uncover new relationships to improve our understanding of the social drivers of health.

## Supporting information

S1 Table Ns for included variables from each wave of data collection. Numbers in this table may differ slightly from those reported in Figs 2–4 because data some respondents (or proxy respondents from the exit files) were collected at multiple waves; we used the first self-report, and then proxy report of information, as detailed in the methods. Please see our code on GitHub for more details (https://github.com/anushavable/Validated-cSES-measures-in-HRS). Data on parent’s educational attainment came from the RAND data files, describe elsewhere.
(DOCX)

S2 Table Father occupation categories. The 1980 census occupation codes had 17 categories, which were used for the following HRS waves: 1996 core, 1998 HRS, 1998 exit, 2000 core, 2000 exit, 2002 core, 2004 core. The 2000 and 2010 census occupation codes had 25 categories, which were used for the following HRS waves: 2006 core, 2006 exit, 2008 exit, 2010 core.
(DOCX)

S3 Table Human capital appendix. Values below 8 were recoded to 8 yearsValues below 0 were recoded to 0 yearsValues greater than 17 were recoded to 17 years
Single-imputations were performed using the iterative expectation maximization algorithm for maximum likelihood estimation within PROC MI procedure in SAS (Truxillo, 2005) across five different subgroups: a) any missing data on education, b) mothers with < 8 years of education (AHEAD coded at 7.5), c) fathers with < 8 years of education (AHEAD coded as 7.5), d) mothers with ≥ 8 years of education (AHEAD coded as 8.5), and e) fathers with ≥ 8 years of education (AHEAD coded as 8.5). All imputation models included birth year, race (Non-Hispanic White (ref), Non-Hispanic Black, Hispanic), gender, birth place (southern, foreign), childhood health (excellent (ref), very good, good, fair, poor), and the following social variables which were significantly correlated with either parents education (operationalization described in main paper text, dummy variables created for categorical variables): father’s occupation, self-reported family SES, moved for financial reasons, received financial help from relatives, father’s unemployment status, mother’s employment status, if the respondent lived with their grandparents, if the respondent didn’t live with their mother, if the respondent didn’t live with their father, amount the respondent’s mother taught them about life, amount of time and attention the respondent received from their mother, and the amount of effort the respondent’s mother put in their upbringing. The imputation model for mother’s education when coded as 7.5 in AHEAD would not converge when all the above variables were included, so these imputations included the following variables that were significantly correlated with mother’s years of education less than 8 years: birth year, race, gender, birth place, childhood health, father’s occupation, self-reported family SES, moved for financial reasons, if the respondent lived with their grandparents, if the respondent didn’t live with their father, and the amount of effort the respondent’s mother put in their upbringing. Imputed values that were outside the expected range were recoded to either the minimum or maximum value for that range.
(DOCX)

S4 Table Factor loadings from the exploratory factor analysis for the retained variables. (DOCX)

S5 Table Reliability of the cSC scale with items serially excluded. The low internal consistency of the cSC scale was not due to any one variable. We believe the low internal consistency is due relatively few questions and dichotomous response options for several items.
(DOCX)

S6 Table Reliability of the cFC scales with items serially excluded. The low internal consistency of the cFC scale was not due to any one variable. We believe the low internal consistency is due to a combination of the following: 1) subjective (i.e. reporting family was “pretty well off”) rather than objective assessments (i.e. renting or owning home, number of bedrooms, number of bathrooms, etc.), 2) relatively few questions on childhood financial capital, and 3) dichotomous response options for many of the included items, reducing precision.
(DOCX)

S7 Table Distribution of social variables for individuals included and excluded by Hargrove operationalization of cSES. * test of equal variance indicated the variances in the two groups were not statistically different, therefore the pooled p-value is displayed rather than the Satterhwaite. The Hargrove operationalization excluded individuals who were more socially disadvantaged; that is, individuals who were born earlier, more likely to be minorities, born in the south or abroad, those who experienced worse childhood health, and those grew up in environments with lower human capital, financial capital and social capital were excluded from the Hargrove analysis.
(DOCX)

S8 Table Means of individuals in the complete case, achievable N, and excluded from the complete case but included in the achievable N analysis. Socially vulnerable individuals excluded from the complete case analysis had more depressive symptoms, pushing the relationship between average financial resources and number of depressive symptoms from a positive relationship in the complete case analysis (contrary to the literature) to a null relationship in the achievable N analysis, which is more consistent with the literature.
(DOCX)

S1 Fig Hypothesized structure for the childhood social capital and financial capital scales, and the childhood human capital index. Based on variables in the HRS data set, we hypothesized the financial capital scale had one factor, the childhood social capital scale had two factors, and we operationalized the childhood human capital index as consisting of mothers and father’s educational attainment.
(DOCX)

S2 Fig Nested family structure questions in experimental module (N = 735) to create the “number of parents” item. This operationalization may lead to some misclassification of categories 1 and 3 because respondents whose parents died may have lived with a stepparent, but were not asked this question. However, due to the other items in the family structure factor / social capital latent variable, including if the respondent lived with their mother or father, the factor analysis should produce appropriate factor scores. Alternative options, such as collapsing this variable into dichotomous response options (i.e. lived with both biological parents vs. did not live with both biological parents) would lead to a lack of co-variation in the “number of parents” and the “grew up without a mother” / “grew up without a father” which is not permitted in factor analysis.
(DOCX)

S3 Fig Distribution of validated measures. None of the validated scales are normally distributed; notably, most of the observations for the social capital scale are at the upper end of the distribution. The combined cSES index has a slightly longer left tail than right tail.
(DOCX)

S4 Fig Scatterplot of average financial resources. The circles represent individuals included in the achievable N analysis but excluded from the complete case analysis; the hashtags represent individuals included in the complete case analysis. This figure shows that the complete case analysis excluded individuals with low average financial resources.
(DOCX)
