# Supplementary material for: Validation of a theoretically motivated approach to measuring childhood socioeconomic circumstances in the Health and Retirement Study
Source: PLoS One. 2017 Oct 13;12(10):e0185898. doi: 10.1371/journal.pone.0185898 (PMC5640422; doi:10.1371/journal.pone.0185898)
Supplement: S1 Table — Numbers in this table may differ slightly from those reported in Figs 2–4 because data some respondents (or proxy respondents from the exit files) were collected at multiple waves; we used the first self-report, and then proxy report of information, as detailed in the methods. Please see our code on GitHub for more details (https://github.com/anushavable/Validated-cSES-measures-in-HRS). Data on parent’s educational attainment came from the RAND data files, describe elsewhere. (DOCX) [file pone.0185898.s001.docx]

| **Variable** | **1996** | | **1998** | | **2000** | | **2002** | | **2004** | | **2006** | | **2008** | | | **2010** |
| --- | --- | --- | --- | --- | --- | --- | --- | --- | --- | --- | --- | --- | --- | --- | --- | --- |
| **Social capital** | **Core** | **Exit** | **Core** | **Exit** | **Core** | **Exit** | **Core** | **Exit** | **Core** | **Exit** | **Core** | **Exit** | **Core** | **Leave behind** | **Exit** | **Core** |
| Mother Effort into upbringing |  |  |  |  |  |  |  |  |  |  |  |  |  | 6647 |  |  |
| Mother time and attention |  |  |  |  |  |  |  |  |  |  |  |  |  | 6649 |  |  |
| Mother taught about life |  |  |  |  |  |  |  |  |  |  |  |  |  | 6419 |  |  |
| Number of parent figures | 735 |  |  |  |  |  |  |  |  |  |  |  |  |  |  |  |
| Live with grandparents | 735 |  |  |  | 19464 | 1223 | 931 | 157 | 3558 | 27 | 278 | 17 | 173 |  | 11 | 156 |
| Didn't live with mother |  |  |  |  |  |  |  |  |  |  | 18343 | 1148 | 705 |  | 120 | 415 |
| Didn't live with father |  |  | 21052 | 1001 | 699 |  | 433 |  | 3499 |  | 236 | 11 | 155 |  |  | 152 |
|  |  |  |  |  |  |  |  |  |  |  |  |  |  |  |  |  |
| **Financial capital** |  |  |  |  |  |  |  |  |  |  |  |  |  |  |  |  |
| Self-rated financial status  (5 categories) | 733 |  |  |  |  |  |  |  |  |  |  |  |  |  |  |  |
| Self-rated financial status  (3 categories) |  |  | 21308 |  | 714 |  | 450 | 70 | 3556 | 17 | 239 | 12 | 157 |  | 6 | 155 |
| Father's occupation | 669 |  | 18087 | 882 | 613 | 111 | 366 |  | 183 |  | 201 | 10 |  |  | 6 | 125 |
| Father unemployed for a long time | 697 |  | 21052 | 1001 | 699 | 82 | 433 | 47 | 3499 | 23 | 236 | 11 | 155 |  | 6 | 152 |
| Received financial help from relatives | 720 |  | 21019 |  | 963 |  | 439 | 59 | 3486 | 26 | 233 | 12 | 151 |  | 6 | 152 |
| Family moved for financial reasons | 728 |  | 21192 |  | 701 |  | 452 | 66 | 3540 | 30 | 242 | 12 | 154 |  | 6 | 156 |
| Family declare bankruptcy | 725 |  |  |  |  |  |  |  |  |  |  |  |  |  |  |  |
| Family lost business | 720 |  |  |  |  |  |  |  |  |  |  |  |  |  |  |  |

S1 Table. Ns for included variables from each wave of data collection
