# Supplementary material for: Validation of a theoretically motivated approach to measuring childhood socioeconomic circumstances in the Health and Retirement Study
Source: PLoS One. 2017 Oct 13;12(10):e0185898. doi: 10.1371/journal.pone.0185898 (PMC5640422; doi:10.1371/journal.pone.0185898)
Supplement: S2 Table — The 1980 census occupation codes had 17 categories, which were used for the following HRS waves: 1996 core, 1998 HRS, 1998 exit, 2000 core, 2000 exit, 2002 core, 2004 core. The 2000 and 2010 census occupation codes had 25 categories, which were used for the following HRS waves: 2006 core, 2006 exit, 2008 exit, 2010 core. (DOCX) [file pone.0185898.s002.docx]

S2 Table. Father occupation categories

| **Ordinal Factor Analysis Variable** | | **1980 census occupation codes** | **1980 occupation titles** | **2000 census occupation codes** | **2000 occupation titles** | **2010 census occupation codes** | **2010 occupation titles** |
| --- | --- | --- | --- | --- | --- | --- | --- |
| 1 | Executives & managers | 003 - 037 | Managerial specialty operation | 000 - 044 | Management occupations | 010 - 043 | Management Occupations |
|  |  |  |  | 050 - 073 | Business operations specialists | 050 - 095 | Business and Financial Operations Occupations |
|  |  |  |  | 080 - 095 | Financial specialists |  |  |
| 2 | Professional specialty | 043 - 235 | Professional specialty operation and technical support | 100 - 124 | Computer and mathematical occupations | 100 - 124 | Computer and mathematical occupations |
|  |  |  |  | 130 - 156 | Architecture and engineering occupations | 130 - 156 | Architecture and engineering occupations |
|  |  |  |  | 160 - 196 | Life, physical and social science occupations | 160 - 196 | Life, physical, and social science occupations |
|  |  |  |  | 200 - 206 | Community and social services occupations | 200 - 206 | Community and social service occupations |
|  |  |  |  | 210 - 215 | Legal occupations | 210 - 216 | Legal occupations |
|  |  |  |  | 220 - 255 | Education, training, and library occupations | 220 - 255 | Education, training, and library occupations |
|  |  |  |  | 260 - 296 | Arts, design, entertainment, sports, and media occupations | 260 - 296 | Arts, design, entertainment, sports, and media occupations |
|  |  |  |  | 300 - 354 | Healthcare practitioners and technical occupations | 300 - 354 | Healthcare practitioners and technical occupations |
|  |  | 445 - 447 | Health services | 360 - 365 | Healthcare Support Occupations | 360 - 365 | Healthcare support occupations |
| 3 | Sales & admin | 243 - 285 | Sales | 470 - 496 | Sales occupations | 470 - 496 | Sales and related occupations |
|  |  | 303 - 389 | Clerical, administration support | 500 - 593 | Office and administrative support occupations | 500 - 594 | Office and administrative support occupations |
| 4 | Protection services & armed forces | 413 - 427 | Service: protection | 370 - 395 | Protective service occupations | 370 - 395 | Protective service occupations |
|  |  | 900 | Members of the Armed Forces | 980 - 985 | Military specific occupations | 980 - 983 | Military Specific Occupations |
| 5 | Cleaning, building, food prep, and personal services | 403 - 407 | Service: private household, cleaning and building services | 420 - 425 | Building and grounds cleaning and maintenance | 420 - 425 | Building and grounds cleaning and maintenance occupations |
|  |  | 433 - 444 | Service: food preparation | 400 - 416 | Food preparation and serving occupations | 400 - 416 | Food preparation and service related occupations |
|  |  | 448 - 469 | Personal services | 430 - 465 | Personal care and service occupations | 430 - 465 | Personal care and service occupations |
| 6 | Production, construction, and operation occupations | 473 - 499 | Farming, forestry, fishing | 600 - 613 | Farming, fishing, and forestry occupations | 600 - 613 | Farming, fishing, and forestry occupations |
|  |  | 503 - 549 | Mechanics and repair | 700 - 762 | Installation, maintenance, and repair workers | 700 - 763 | Installation, maintenance, and repair occupations |
|  |  | 553 - 617 | Construction trade and extractors | 620 - 676 | Construction trades | 620 - 694 | Construction and extraction occupations |
|  |  |  |  | 680 - 694 | Extraction workers |  |  |
|  |  | 633 - 699 | Precision production | 770 - 896 | Production occupations | 770 - 896 | Production occupations |
|  |  | 703 - 799 | Operators: machine | 900 - 975 | Transportation and material moving occupations | 900 - 975 | Transportation and material moving occupations |
|  |  | 803 - 859 | Operators: transport, etc. |  |  |  |  |
|  |  | 863 - 889 | Operators: handlers, etc. |  |  |  |  |
|  |  |  |  |  |  |  |  |
|  | | | | | | | |
