# Supplementary material for: Validation of a theoretically motivated approach to measuring childhood socioeconomic circumstances in the Health and Retirement Study
Source: PLoS One. 2017 Oct 13;12(10):e0185898. doi: 10.1371/journal.pone.0185898 (PMC5640422; doi:10.1371/journal.pone.0185898)
Supplement: S3 Table — Values below 8 were recoded to 8 yearsValues below 0 were recoded to 0 yearsValues greater than 17 were recoded to 17 years Single-imputations were performed using the iterative expectation maximization algorithm for maximum likelihood estimation within PROC MI procedure in SAS (Truxillo, 2005) across five different subgroups: a) any missing data on education, b) mothers with < 8 years of education (AHEAD coded at 7.5), c) fathers with < 8 years of education (AHEAD coded as 7.5), d) mothers with ≥ 8 years of education (AHEAD coded as 8.5), and e) fathers with ≥ 8 years of education (AHEAD coded as 8.5). All imputation models included birth year, race (Non-Hispanic White (ref), Non-Hispanic Black, Hispanic), gender, birth place (southern, foreign), childhood health (excellent (ref), very good, good, fair, poor), and the following social variables which were significantly correlated with either parents education (operationalization described in main paper text, dummy variables created for categorical variables): father’s occupation, self-reported family SES, moved for financial reasons, received financial help from relatives, father’s unemployment status, mother’s employment status, if the respondent lived with their grandparents, if the respondent didn’t live with their mother, if the respondent didn’t live with their father, amount the respondent’s mother taught them about life, amount of time and attention the respondent received from their mother, and the amount of effort the respondent’s mother put in their upbringing. The imputation model for mother’s education when coded as 7.5 in AHEAD would not converge when all the above variables were included, so these imputations included the following variables that were significantly correlated with mother’s years of education less than 8 years: birth year, race, gender, birth place, childhood health, father’s occupation, self-reported family SES, moved for financial reasons, if the respondent lived with their grandpare [file pone.0185898.s003.docx]

S3 Table. Human capital appendix

|  | Before Imputations | | | | Imputation Subgroups | | | | | | | | | | | | After Imputations | | | |
| --- | --- | --- | --- | --- | --- | --- | --- | --- | --- | --- | --- | --- | --- | --- | --- | --- | --- | --- | --- | --- |
|  |  | | | | AHEAD coded as 7.5 | | | | AHEAD coded as 8.5 | | | | Missing Data | | | |  | | | |
|  | N | Mean | Min | Max | N | Mean | Min | Max | N | Mean | Min | Max | N | Mean | Min | Max | N | Mean | Min | Max |
| Mother’s education | 19847 | 9.58 | 0 | 17.0 | 3375 | 4.46 | 1.48 | 6.26 | 3848 | 10.1 | 7.95^a^ | 13.99 | 4099 | 8.34 | 0.82 | 15.20 | 31169 | 8.93 | 0 | 17 |
| Father’s education | 18595 | 9.26 | 0 | 17.0 | 3638 | 4.15 | 1.51 | 5.74 | 3572 | 10.23 | 7.20^a^ | 16.07 | 5364 | 8.11 | -0.09^b^ | 17.25^c^ | 31169 | 8.58 | 0 | 17 |
