# Supplementary material for: Validation of a theoretically motivated approach to measuring childhood socioeconomic circumstances in the Health and Retirement Study
Source: PLoS One. 2017 Oct 13;12(10):e0185898. doi: 10.1371/journal.pone.0185898 (PMC5640422; doi:10.1371/journal.pone.0185898)
Supplement: S4 Table — (DOCX) [file pone.0185898.s004.docx]

S4 Table. Factor loadings from the exploratory factor analysis for the retained variables

|  |  | **Factor Loading** | |
| --- | --- | --- | --- |
| **Scale** | **Item** | **Factor 1** | **Factor 2** |
| **Childhood social capital scale** | Mother amount of time & attention | 0.92 |  |
|  | Mother effort into upbringing | 0.97 |  |
|  | Mother taught about life | 0.87 |  |
|  | Number of parents |  | 0.96 |
|  | Didn’t live with mother |  | 0.45 |
|  | Didn’t live with father |  | 0.78 |
|  | Lived with grandparents |  | 0.34 |
| **Childhood financial capital scale** | Self-rated childhood SES (3 categories) | 0.86 |  |
|  | Self-rated childhood SES (5 categories) | 0.83 |  |
|  | Father’s occupation | 0.50 |  |
|  | Father unemployed for a long time | -0.43 |  |
|  | Family declared bankruptcy |  | 0.89 |
|  | Family lost business |  | 0.96 |
|  | Family moved for financial reasons |  | 0.58 |
|  | Received financial help from relatives |  | 0.64 |
