# Supplementary material for: Validation of a theoretically motivated approach to measuring childhood socioeconomic circumstances in the Health and Retirement Study
Source: PLoS One. 2017 Oct 13;12(10):e0185898. doi: 10.1371/journal.pone.0185898 (PMC5640422; doi:10.1371/journal.pone.0185898)
Supplement: S5 Table — The low internal consistency of the cSC scale was not due to any one variable. We believe the low internal consistency is due relatively few questions and dichotomous response options for several items. (DOCX) [file pone.0185898.s005.docx]

S5 Tables. Reliability of the cSC scale with items serially excluded

|  | | Standardized Cronbach’s Alpha |
| --- | --- | --- |
| Childhood social capital scale | | 0.63 |
| Variable excluded | |  |
|  | Mother effort into upbringing | 0.53 |
|  | Mother time & attention | 0.53 |
|  | Mother taught about life | 0.54 |
|  | Number of parents | 0.64 |
|  | Lived with grandparents | 0.67 |
|  | Did not grow up with mother | 0.64 |
|  | Did not grow up with father | 0.65 |
