# Supplementary material for: Validation of a theoretically motivated approach to measuring childhood socioeconomic circumstances in the Health and Retirement Study
Source: PLoS One. 2017 Oct 13;12(10):e0185898. doi: 10.1371/journal.pone.0185898 (PMC5640422; doi:10.1371/journal.pone.0185898)
Supplement: S6 Table — The low internal consistency of the cFC scale was not due to any one variable. We believe the low internal consistency is due to a combination of the following: 1) subjective (i.e. reporting family was “pretty well off”) rather than objective assessments (i.e. renting or owning home, number of bedrooms, number of bathrooms, etc.), 2) relatively few questions on childhood financial capital, and 3) dichotomous response options for many of the included items, reducing precision. (DOCX) [file pone.0185898.s006.docx]

S6 Table. Reliability of the cFC scales with items serially excluded

|  | | Standardized Cronbach’s Alpha |
| --- | --- | --- |
| Childhood financial capital scale | | 0.63 |
| Variable excluded | |  |
|  | Father’s occupation | 0.65 |
|  | Self-reported cSES, 3-cat | 0.60 |
|  | Self-reported cSES, 5-cat | 0.58 |
|  | Father unemployed for a long time | 0.61 |
|  | Received financial help from relatives | 0.58 |
|  | Moved for financial reasons | 0.55 |
|  | Family lost business | 0.61 |
|  | Family declared bankruptcy | 0.55 |
