# Supplementary material for: Validation of a theoretically motivated approach to measuring childhood socioeconomic circumstances in the Health and Retirement Study
Source: PLoS One. 2017 Oct 13;12(10):e0185898. doi: 10.1371/journal.pone.0185898 (PMC5640422; doi:10.1371/journal.pone.0185898)
Supplement: S7 Table — * test of equal variance indicated the variances in the two groups were not statistically different, therefore the pooled p-value is displayed rather than the Satterhwaite. The Hargrove operationalization excluded individuals who were more socially disadvantaged; that is, individuals who were born earlier, more likely to be minorities, born in the south or abroad, those who experienced worse childhood health, and those grew up in environments with lower human capital, financial capital and social capital were excluded from the Hargrove analysis. (DOCX) [file pone.0185898.s007.docx]

S7 Table. Distribution of social variables for individuals included and excluded by Hargrove operationalization of cSES

| Variable | | Mean for individuals Hargrove operationalized included | Mean for individuals Hargrove operationalized excluded | Satterthwaite  p-value |
| --- | --- | --- | --- | --- |
| Demographics | |  |  |  |
|  | Birth year | 1937.4 | 1930.6 | <0.0001 |
|  | Non-Hispanic White | 0.79 | 0.71 | < 0.0001 |
|  | Non-Hispanic Black | 0.11 | 0.17 | <0.0001 |
|  | Hispanic | 0.08 | 0.09 | <0.0001 |
|  | Other Race | 0.02 | 0.03 | 0.0622 |
|  | Southern born | 0.34 | 0.36 | <0.0001* |
|  | Foreign born | 0.09 | 0.12 | <0.0001 |
| Childhood health | |  |  |  |
|  | Excellent | 0.54 | 0.47 | <0.0001* |
|  | Very good | 0.25 | 0.26 | 0.0083 |
|  | Good | 0.16 | 0.20 | <0.0001 |
|  | Fair | 0.05 | 0.05 | 0.0021 |
|  | Poor | 0.01 | 0.02 | 0.0320 |
| Validated measures | |  |  |  |
|  | Maternal investment | -0.01 | -0.07 | <0.0001* |
|  | Family structure | 0.01 | -0.05 | <0.0001 |
|  | Average financial resources | 0.01 | -0.04 | <0.0001* |
|  | Financial instability | 0.01 | 0.09 | <0.0001* |
|  | Mother’s education | 9.62 | 8.48 | <0.0001 |
|  | Father’s education | 9.20 | 8.18 | <0.0001 |
