# Supplementary material for: Validation of a theoretically motivated approach to measuring childhood socioeconomic circumstances in the Health and Retirement Study
Source: PLoS One. 2017 Oct 13;12(10):e0185898. doi: 10.1371/journal.pone.0185898 (PMC5640422; doi:10.1371/journal.pone.0185898)
Supplement: S8 Table — Socially vulnerable individuals excluded from the complete case analysis had more depressive symptoms, pushing the relationship between average financial resources and number of depressive symptoms from a positive relationship in the complete case analysis (contrary to the literature) to a null relationship in the achievable N analysis, which is more consistent with the literature. (DOCX) [file pone.0185898.s008.docx]

S8 Table. Means of individuals in the complete case, achievable N, and excluded from the complete case but included in the achievable N analysis.

| Variable | Included in complete case  (N = 7783) | Included in achievable N  (N = 14,166) | Excluded from the complete case but included in the achievable N analysis  (N = 6,383) |
| --- | --- | --- | --- |
| Mean value of average financial resources | 0.01 | -0.02 | -0.05 |
| Mean number of depressive symptoms | 1.22 | 1.41 | 1.64 |
