# Supplementary material for: Validation of a theoretically motivated approach to measuring childhood socioeconomic circumstances in the Health and Retirement Study
Source: PLoS One. 2017 Oct 13;12(10):e0185898. doi: 10.1371/journal.pone.0185898 (PMC5640422; doi:10.1371/journal.pone.0185898)
Supplement: S1 Fig — Based on variables in the HRS data set, we hypothesized the financial capital scale had one factor, the childhood social capital scale had two factors, and we operationalized the childhood human capital index as consisting of mothers and father’s educational attainment. (DOCX) [file pone.0185898.s009.docx]

Childhood social capital

Didn’t live with mother

Number of parent figures

Live with grandparents?

Mother amount of time & attention

Mother taught about life

Mother effort into upbringing

Maternal Investment

Family Structure

Didn’t live with father

Family lose business?

Family self-rated financial status (5 categories)

Family self-rated financial status (3 categories)

Father’s occupation

Father unemployed for a long time

Family moved for financial reasons

Received financial help from relatives

Mother worked

Childhood financial capital

Family declare bankruptcy?

Childhood human capital

Mother’s years of education

Father’s years of education

S1 Fig. Hypothesized structure for the childhood social capital and financial capital scales, and the childhood human capital index
