# Supplementary material for: Validation of a theoretically motivated approach to measuring childhood socioeconomic circumstances in the Health and Retirement Study
Source: PLoS One. 2017 Oct 13;12(10):e0185898. doi: 10.1371/journal.pone.0185898 (PMC5640422; doi:10.1371/journal.pone.0185898)
Supplement: S2 Fig — This operationalization may lead to some misclassification of categories 1 and 3 because respondents whose parents died may have lived with a stepparent, but were not asked this question. However, due to the other items in the family structure factor / social capital latent variable, including if the respondent lived with their mother or father, the factor analysis should produce appropriate factor scores. Alternative options, such as collapsing this variable into dichotomous response options (i.e. lived with both biological parents vs. did not live with both biological parents) would lead to a lack of co-variation in the “number of parents” and the “grew up without a mother” / “grew up without a father” which is not permitted in factor analysis. (DOCX) [file pone.0185898.s010.docx]

S2 Fig. Nested family structure questions in experimental module (N = 735) to create the “number of parents” item

Live with both parents?

(a) Yes, N = 539

(b) Don’t know / refused, N = 3

No, N = 193

Why not both parents?

Parents divorced / separated, N = 84

Live with stepparent?

(c) Parent died, N = 77

(d) Other reason besides death or divorce, N = 29

(e) Don’t know / refused, N = 3

(f) Yes, N = 32

(g) No, N = 51

(h) Missing, N = 1

**Terminal Response Options**

**Nested Questions**

Ordinal categorization of response options for nested family structure questions for use in factor analysis

| Ordinal category | Question | N |
| --- | --- | --- |
| 0 | Lived with both biological parents (a) | 539 |
| 1 | Lived with two parents, one biological (f) | 32 |
| 2 | Had two parents, but parents did not live together (d, g) | 80 |
| 3 | Did not have two parents (b, c, e, h) | 84 |
