# Supplementary material for: Validation of a theoretically motivated approach to measuring childhood socioeconomic circumstances in the Health and Retirement Study
Source: PLoS One. 2017 Oct 13;12(10):e0185898. doi: 10.1371/journal.pone.0185898 (PMC5640422; doi:10.1371/journal.pone.0185898)
Supplement: S3 Fig — None of the validated scales are normally distributed; notably, most of the observations for the social capital scale are at the upper end of the distribution. The combined cSES index has a slightly longer left tail than right tail. (DOCX) [file pone.0185898.s011.docx]

S3 Fig. Distribution of validated measures

| **Childhood human capital index** | **Childhood financial capital scale** |
| --- | --- |
| 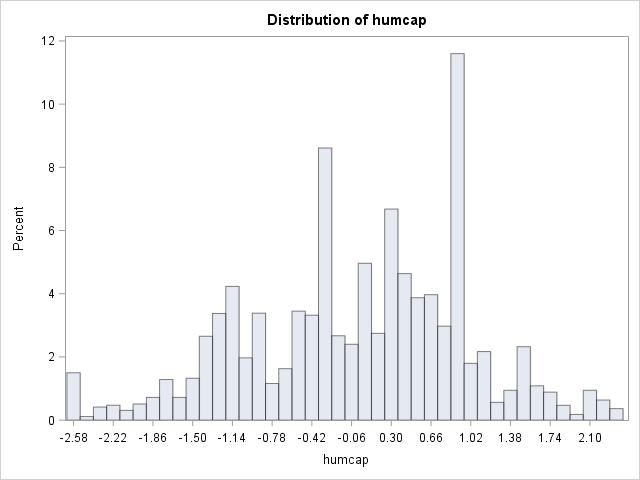 | 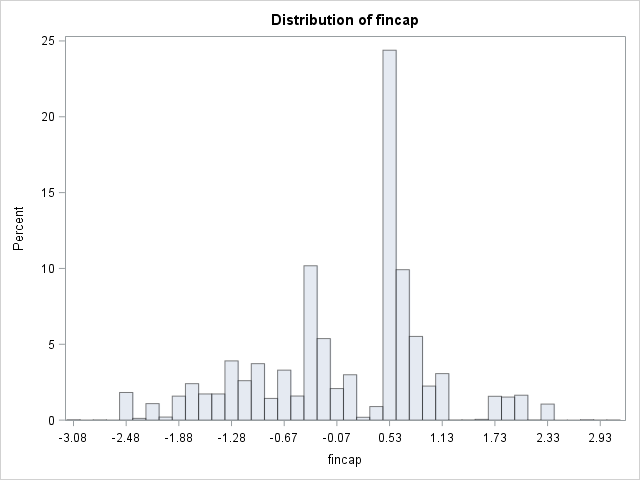 |
| **Childhood social capital scale** | **Combined cSES index** |
| 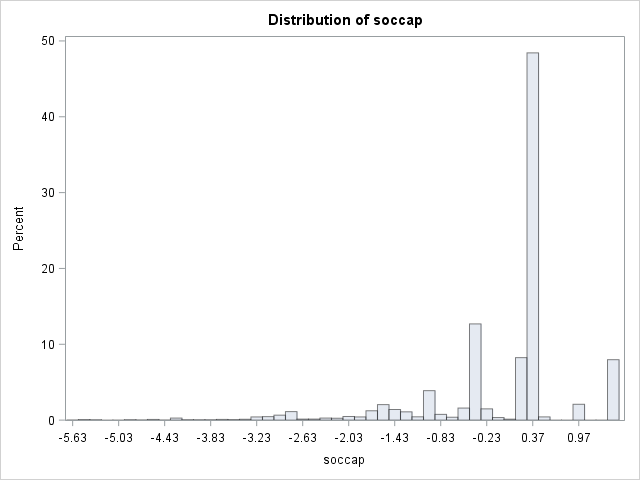 | 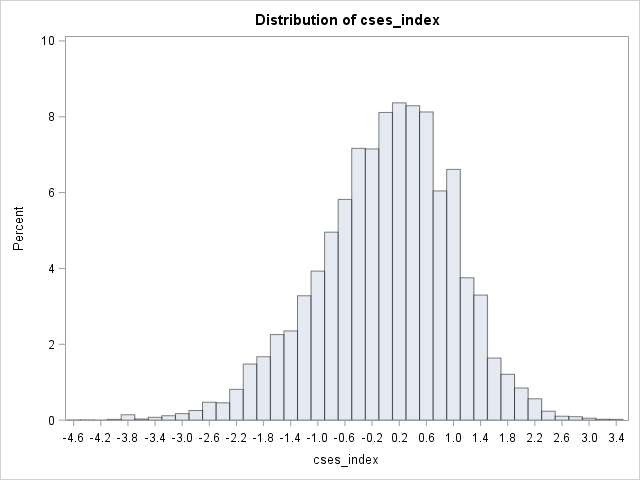 |
