# Supplementary figures and images for: Validation of a theoretically motivated approach to measuring childhood socioeconomic circumstances in the Health and Retirement Study
Source: PLoS One. 2017 Oct 13;12(10):e0185898. doi: 10.1371/journal.pone.0185898 (PMC5640422; doi:10.1371/journal.pone.0185898)

S4 Fig. Scatterplot of average financial resources.


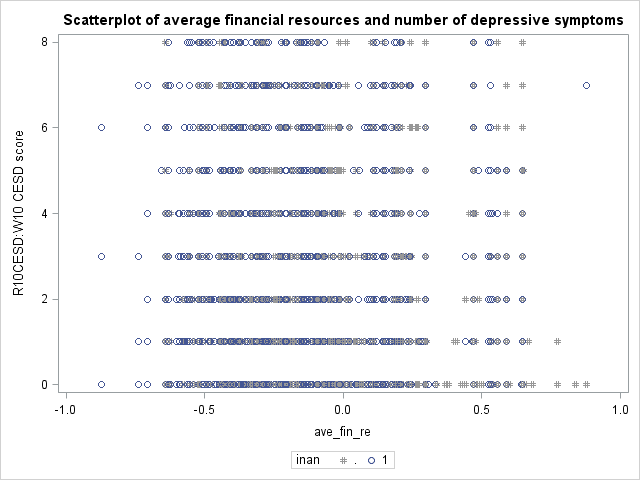

Supplement: S4 Fig — The circles represent individuals included in the achievable N analysis but excluded from the complete case analysis; the hashtags represent individuals included in the complete case analysis. This figure shows that the complete case analysis excluded individuals with low average financial resources. (DOCX) [file pone.0185898.s012.docx]
